# Plastid Phylogenomics Provide Evidence to Accept Two New Members of *Ligusticopsis* (Apiaceae, Angiosperms)

**DOI:** 10.3390/ijms24010382

**Published:** 2022-12-26

**Authors:** Changkun Liu, Jiaojiao Deng, Renxiu Zhou, Boni Song, Songdong Zhou, Xingjin He

**Affiliations:** Key Laboratory of Bio-Resources and Eco-Environment of Ministry of Education, College of Life Sciences, Sichuan University, Chengdu 610065, China

**Keywords:** Apiaceae, *Peucedanum*, *Ligusticopsis*, new combination, plastome, phylogenomics

## Abstract

*Peucedanum nanum* and *P. violaceum* are recognized as members of the genus *Peucedanum* because of their dorsally compressed mericarps with slightly prominent dorsal ribs and narrowly winged lateral ribs. However, these species are not similar to other *Peucedanum* taxa but resemble *Ligusticopsis* in overall morphology. To check the taxonomic positions of *P. nanum* and *P. violaceum*, we sequenced their complete plastid genome (plastome) sequences and, together with eleven previously published *Ligusticopsis* plastomes, performed comprehensively comparative analyses. The thirteen plastomes were highly conserved and similar in structure, size, GC content, gene content and order, IR borders, and the patterns of codon bias, RNA editing, and simple sequence repeats (SSRs). Nevertheless, twelve mutation hotspots (*mat*K, *ndh*C, *rps*15, *rps*8, *ycf*2, *ccs*A-*ndh*D, *pet*N-*psb*M, *psb*A-*trn*K, *rps*2-*rpo*C2, *rps*4-*trn*T, *trn*H-*psb*A, and *ycf*2-*trn*L) were selected. Moreover, both the phylogenetic analyses based on plastomes and on nuclear ribosomal DNA internal transcribed spacer (ITS) sequences robustly supported that *P. nanum* and *P. violaceum* nested in *Ligusticopsis*, and this was further confirmed by the morphological evidence. Hence, transferring *P. nanum* and *P. violaceum* into *Ligusticopsis* genus is reasonable and convincing, and two new combinations are presented.

## 1. Introduction

*Ligusticopsis* Leute, a small flowering plant genus of Apiaceae, was established by Gerfried Horand Leute in 1969 with *L. rechingeriana* Leute as the type species, recognizable by conspicuous calyx teeth and strongly dorsally flattened mericarps with numerous vallecular vittae [1]. However, these characteristics can also be detected in some *Ligusticum* L. members [2]; therefore, the morphological delimitation between *Ligusticopsis* and *Ligusticum* is historically unclear. Furthermore, several *Ligusticopsis* species described by Leute do not have prominent calyx teeth [3], which further blurred the boundaries of this genus. Hence, the genus *Ligusticopsis* has been merged into the genus *Ligusticum* by some authors [2,4,5,6]. However, Li et al. [7] recently confirmed that the genus *Ligusticopsis* is a natural unit based on molecular and morphological evidence, and confirmed the presence of nine “true *Ligusticopsis* species”. Subsequently, the phylogenetic analyses based on plastome data performed by Ren et al. [8] also recovered *Ligusticopsis* as a monophyletic group and recognized two additional species. To date, the genus *Ligusticopsis* contains eleven validated species.

**Figure 1:**
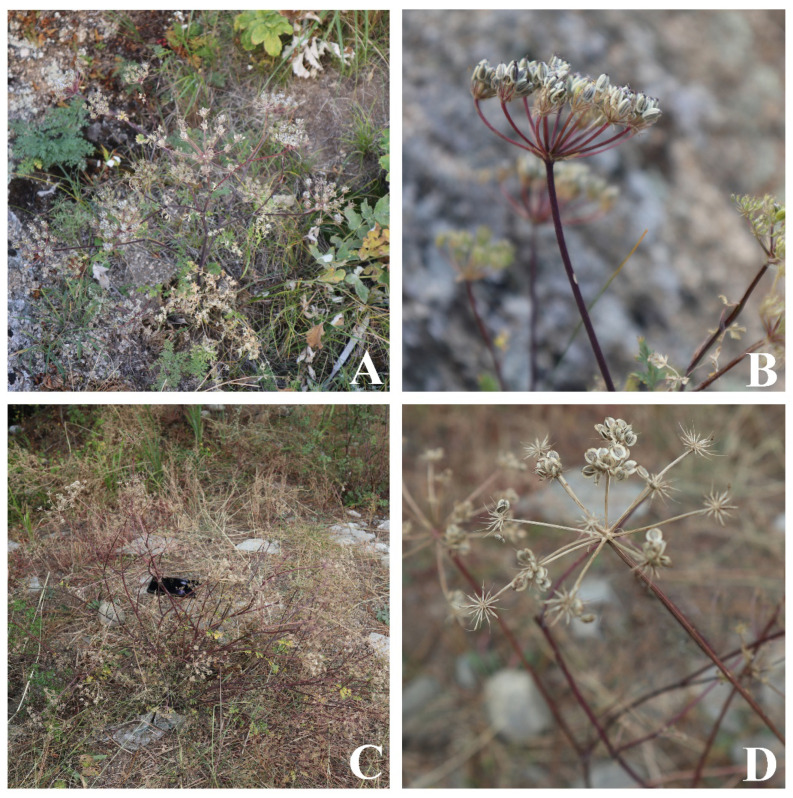
Morphology of *P. nanum* (**A**,**B**) and *P. violaceum* (**C**,**D**).

*Peucedanum nanum* R.H.Shan and M.L.Sheh and *P. violaceum* R.H.Shan and M.L.Sheh are species endemic to China, which grow on dry mountain slopes and in sparse forests or grassy places on riverbanks, respectively [9,10]. Both species are placed in *Peucedanum* L. owing to their dorsally compressed mericarps with slightly prominent dorsal ribs and narrowly winged lateral ribs [11]. However, *P. nanum* and *P. violaceum* are not similar to the type species of *Peucedanum* (*P. officinale* L.) [12] but resemble *Ligusticopsis* species in overall morphology (Figure 1). Additionally, the genus *Peucedanum* is not monophyletic [13,14,15,16,17,18,19], and its taxonomy has faced extreme challenges. Therefore, the taxonomic positions of *P. nanum* and *P. violaceum* need to be re-evaluated.

A robust molecular phylogenetic framework could provide valuable information for resolving the taxonomic positions of *P. nanum* and *P. violaceum*. Unfortunately, molecular data for both species are limited, and these species have not been included in previous phylogenetic studies. Hence, it is necessary to identify molecular markers to investigate the phylogenetic position of *P. nanum* and *P. violaceum*.

The plastid genome (plastome) sequence, possessing highly variable characters, gives us the potential to obtain a robust phylogenetic framework at low taxonomic levels [20,21,22,23,24,25,26,27,28,29]. With the development of next-generation sequencing, plastome sequences have been applied extensively and successfully to resolve the phylogenetic position of taxonomically difficult taxa [8,30,31,32,33,34,35]. In this study, we sequenced and assembled the plastomes of *P. nanum* and *P. violaceum* for the first time. Together with the previously published eleven *Ligusticopsis* plastomes, we carried out comprehensively comparative analyses to reveal the plastome features for *P. nanum*, *P. violaceum*, and *Ligusticopsis* species. Subsequently, we performed phylogenetic analyses based on the plastome data and nuclear ribosomal DNA internal transcribed spacer (ITS) sequences to investigate the phylogenetic positions of *P. nanum* and *P. violaceum*. Finally, by combining evidence from the comparative plastome analyses, molecular phylogeny, and morphology, taxonomic revisions for *P. nanum* and *P. violaceum* were conducted.

## 2. Results

### 2.1. Plastome Features

Illumina sequencing obtained 31,564,816 and 33,651,636 paired-end clean reads for *P. nanum* and *P. violaceum*, respectively (Table S1); among these reads, 1,025,204 and 543,725 reads were mapped to the assemblies, respectively. Based on these data, two high-quality plastomes for *P. nanum* and *P. violaceum* were generated with 1022.351× and 536.171× coverage, respectively.

**Figure 2:**
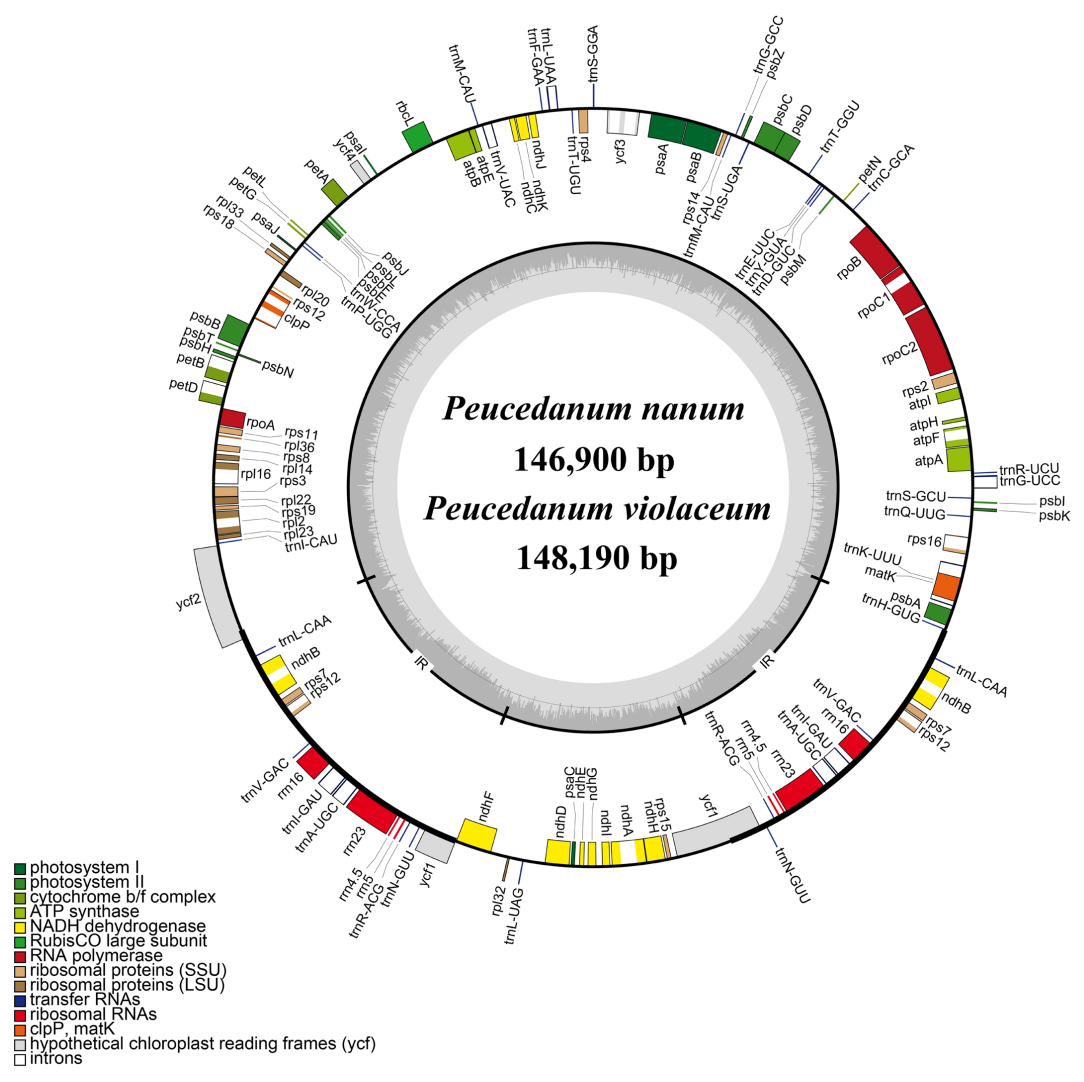
Whole plastome map of *Peucedanum nanum* and *P. violaceum*. Genes shown outside of the circle were transcribed counterclockwise, while those presented inside were transcribed clockwise. The dark gray area of the inner circle shows the GC content of the plastome.

The plastome features of eleven *Ligusticopsis* taxa and two *Peucedanum* species were comprehensively investigated. The overall size ranged from 146,900 bp (*P. nanum*) to 148,633 bp (*L. brachyloba* (Franch.) Leute) in the thirteen plastomes (Table 1). All of them exhibited typical quadripartite structures containing a pair of inverted repeat regions (IRs, 19,056–20,022 bp) divided by a large single-copy region (LSC, 91,480–92,305 bp) and a small single-copy region (SSC, 16,335–17,654 bp) (Table 1, Figure 2). The total GC content of the thirteen plastomes ranged from 37.3% to 37.5%, and 113 unique genes were identified, including 79 protein-coding genes, 30 tRNA genes, and four rRNA genes (Table 1 and Table S2).

**Figure 3:**
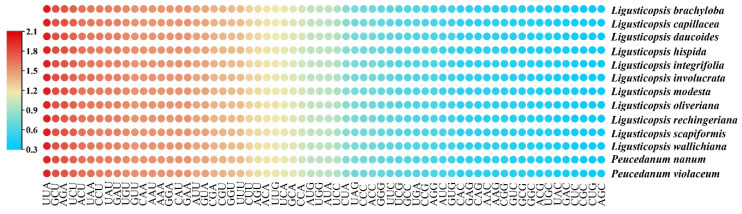
The RSCU values of 79 protein-coding regions for thirteen plastomes. Red represents higher RSCU values, while blue indicates lower RSCU values.

The 79 protein-coding genes typically shared by the thirteen plastomes were extracted and connected for each species. These sequences were 67,566–67,896 bp in length and harbored 22,522–22,632 codons (Table S3). Among these codons, the least number of codons were used to encode the Cys, while the highest number of codons were used to encode the Leu. Additionally, the relative synonymous codon usage (RSCU) values of all codons ranged from 0.34 to 2.00 in the thirteen plastomes (Figure 3). Among them, the RSCU values of 30 codons were greater than 1.00 in all plastomes. All of these codons ended with A/U, except for UUG.

A total of 57–59 potential RNA editing sites were identified in the thirteen plastomes (Table S4). All detected RNA editing sites were Cytosine to Uracil (C-U) conversion, and most of them occurred in the second codon position (43–45) followed by the first codon position (14), but no site was located in the third codon position (Table S5). Moreover, the *ndh*B gene contained the highest number of RNA editing sites (10) in all plastomes (Table S6).

**Figure 4:**
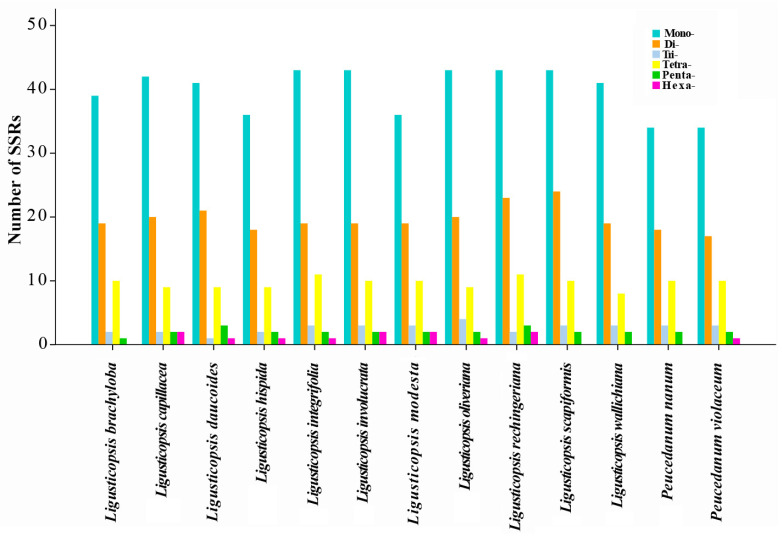
The different repeat types identified in thirteen plastomes.

The total number of simple sequence repeats (SSRs) ranged from 67 to 84 among the thirteen plastomes (Figure 4). Among these, mononucleotide repeats were the most abundant (34–43) followed by dinucleotides (17–24). In addition, bases A and T were dominant for all the identified SSRs in the thirteen plastomes (Table S7).

### 2.2. Plastome Comparison

**Figure 5:**
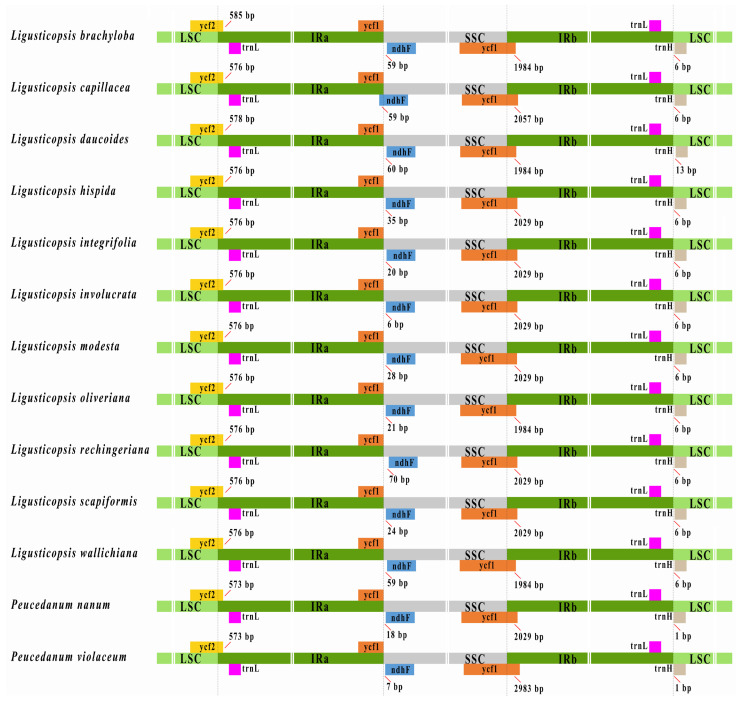
Comparison of IR borders among thirteen plastomes.

The borders between the IR and SC among the thirteen plastomes were compared (Figure 5). The junctions of IRa/LSC and IRb/LSC fell into the *ycf*2 gene and intergenic region of *trn*L-*trn*H, respectively. The borders of IRb/SSC fell into the *ycf*1 gene in all species, whereas the overlap between the *ycf*1 gene and the *ndh*F gene in the IRa/SSC junctions was only detected in *L. capillacea* (H.Wolff) Leute.

**Figure 6:**
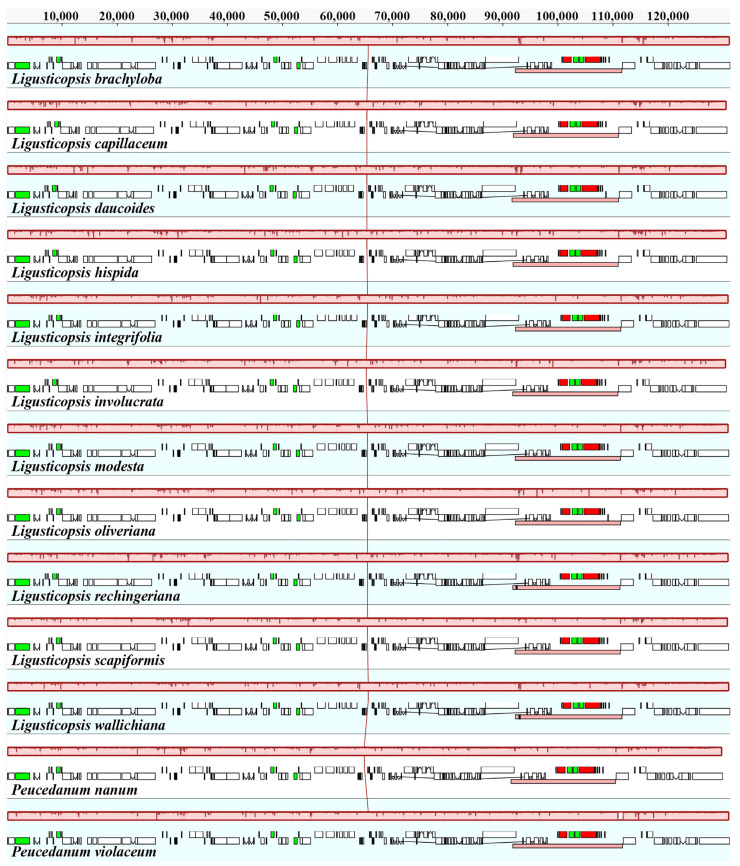
Mauve alignment of thirteen plastomes.

**Figure 7:**
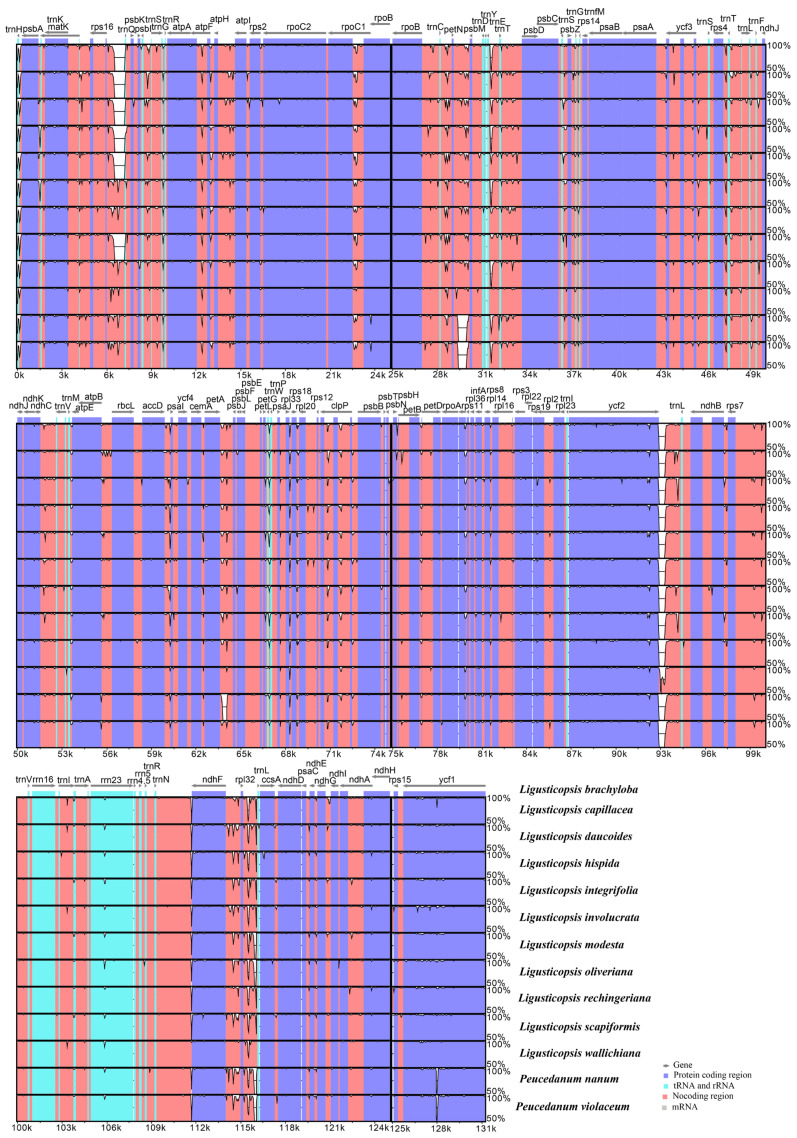
mVISTA alignment for thirteen plastomes.

**Figure 8:**
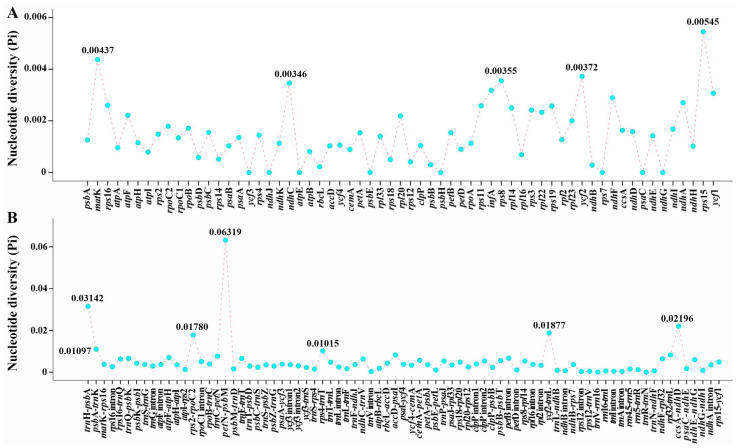
The nucleotide diversity (Pi) values among thirteen plastomes. (**A**) protein-coding regions; (**B**) non-coding and intron regions.

The gene arrangement among the thirteen plastomes was the same (Figure 6), and their sequences showed high similarity with 98.2% pairwise identity (Figure 7). Nevertheless, 12 mutation hotspot regions were identified, including five protein-coding genes (*mat*K, *ndh*C, *rps*15, *rps*8, *ycf*2) that exhibited Pi > 0.00340 and 7 non-coding regions (*ccs*A-*ndh*D, *pet*N-*psb*M, *psb*A-*trn*K, *rps*2-*rpo*C2, *rps*4-*trn*T, *trn*H-*psb*A, *ycf*2-*trn*L) that showed Pi > 0.01000 (Figure 8).

### 2.3. Phylogenetic Analyses

**Figure 9:**
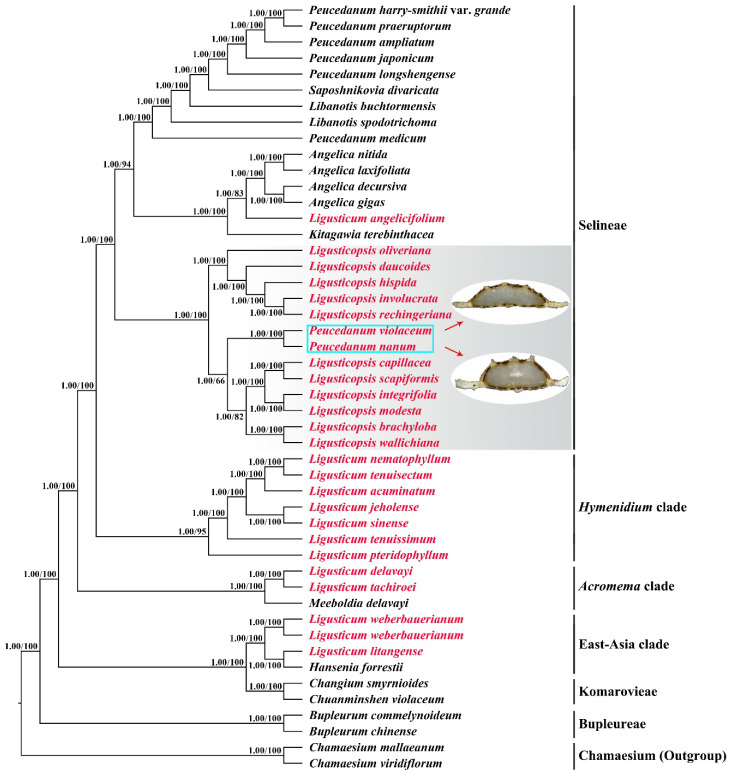
Phylogenetic tree inferred from maximum likelihood (ML) and Bayesian inference (BI) analyses based on plastome data. The numbers indicate Bayesian posterior probabilities (PP) and maximum likelihood bootstrap values (BS), respectively.

The analyses of maximum likelihood (ML) and Bayesian inference (BI) based on the plastome data generated identical tree topologies. As shown in Figure 9, the eleven *Ligusticopsis* taxa, *P. nanum*, and *P. violaceum* clustered as a clade (BI posterior probabilities, PP = 1.00, ML bootstrap values, BS = 100) within Selineae (PP = 1.00, BS = 100), which was clearly distant from other *Ligusticum* taxa. Within this clade, three lineages could be recognized: (1) *L. daucoides* (Franch.) Lavrova ex Pimenov and Kljuykov, *L. hispida* (Franch.) Lavrova and Kljuykov, L. *involucrata* (Franch.) Lavrova, *L. oliveriana* (H.Boissieu) Lavrova, and *L. rechingeriana* formed a clade (PP = 1.00, BS = 100) in which *L. oliveriana* early diverged from the reminders (PP = 1.00, BS = 100) followed by *L. daucoides* (PP = 1.00, BS = 100), and the sub-clade *L. involucrata + L. rechingeriana* (PP = 1.00, BS = 100) sister to *L. hispida* (PP = 1.00, BS = 100); (2) *P. nanum* and *P. violaceum* represented a clade (PP = 1.00, BS = 100); (3) the six remainders constituted another clade (PP = 1.00, BS = 82) in which *L. capillacea + L. scapiformis* (H.Wolff) Leute was sister to *L. integrifolia* (H.Wolff) Leute *+ L. modesta* (Diels) Leute (PP = 1.00, BS = 100) and then clustered with *L. brachyloba + L. wallichiana* (DC.) Pimenov and Kljuykov (PP = 1.00, BS = 82).

Although phylogenetic analyses based on ITS sequences yielded topologies with low support and resolution, the results also indicated that the sister group of *P. nanum* and *P. violaceum* clustered with the *Ligusticopsis* species (PP = 1.00, BS = 97), and this clade was relatively distant from other *Ligusticum* taxa (Figure S1).

## 3. Discussion

### 3.1. Plastome Features

In this study, we conducted comprehensively comparative analyses for the plastomes of *P. nanum, P. violaceum*, and *Ligusticopsis* species. The thirteen plastomes showed typical quadripartite structures, including a pair of inverted repeat regions divided by a large single-copy region and a small single-copy region, which is the same as the other plastomes of Apiaceae [7,8,19,36,37,38,39,40,41]. Although gene loss and rearrangement have been reported in the plastomes of Apiaceae [19,38,39], the gene content and order in the thirteen studied plastomes were identical. All these plastomes also shared similar genomic size, total GC content, and IR borders. Furthermore, the patterns of codon bias, RNA editing sites, and SSR were extremely similar and have also been detected in the plastomes of *Ligusticum* and *Peucedanum* within Apiaceae [19,42]. These results indicated that the thirteen plastomes were highly conserved. Meanwhile, the conserved and similar plastome characters among *P. nanum*, *P. violaceum*, and *Ligusticopsis* species also implied that *P. nanum* and *P. violaceum* may be members of *Ligusticopsis*. 

Although the thirteen plastomes showed high similarity, 12 mutation hotspot regions (*mat*K, *ndh*C, *rps*15, *rps*8, *ycf*2, *ccs*A-*ndh*D, *pet*N-*psb*M, *psb*A-*trn*K, *rps*2-*rpo*C2, *rps*4-*trn*T, *trn*H-*psb*A, *ycf*2-*trn*L) were still identified, which could be used as potential DNA barcodes for species identification and phylogenetic analysis of the *Ligusticopsis* species. Among them, the *mat*K gene and the *trn*H-*psb*A fragment have been suggested as universal DNA barcodes [43,44,45,46], while the *ycf*2 gene and the *pet*N-*psb*M region have been extensively used for phylogenetic analysis [47,48,49,50,51,52,53]. In future studies, the effects of these sequences on species identification and phylogenetic analysis of *Ligusticopsis* will be further validated.

### 3.2. Phylogenetic Inference

Since the establishment of the genus *Ligusticopsis*, its taxonomy has been controversial. Pu [2], Pu and Watson [5], Zhang [4], and Wang et al. [54] did not recognize *Ligusticopsis* as a distinct genus but merged it into *Ligusticum* based on morphological characteristics. However, based on carpoanatomical evidence, Pimenov et al. [55] accepted the establishment of *Ligusticopsis*. Subsequently, Pimenov [56] recognized 18 *Ligusticopsis* species in his checklist of Chinese Umbelliferae based on reviews of the type specimens and morphological evidence. Recently, plastome phylogenetic analyses performed by Li et al. [7] and Ren et al. [8] robustly confirmed the monophyly of *Ligusticopsis*, although limited samples of *Ligusticopsis* and *Ligusticum* were used in both studies. In the present study, twelve *Ligusticum* species and eleven *Ligusticopsis* taxa were included in the phylogenetic analyses. Both the phylogenies based on plastome data and on ITS sequences revealed that eleven *Ligusticopsis* species clustered as a clade and belonged to the Selineae tribe. Although the type species of *Ligusticum (Ligusticum scoticum* L.) was absent in our analyses, the phylogenetic position of this species, located in the *Acronema* Clade, was revealed by a previous study [18], which was obviously distant from the clade formed by the *Ligusticopsis* species. Our results with more extensive taxa sampling provided additional evidence to accept *Ligusticopsis* as a distinct genus.

Additionally, all our phylogenetic analyses based on plastome data and ITS sequences robustly supported that *P. nanum* and *P. violaceum* nested within *Ligusticopsis*. The type species of *Peucedanum* (*P. officinale*) was not included in our analyses; however, a previous study revealed its phylogenetic location was distant from *Ligusticopsis* [18]. These results implied that *P. nanum* and *P. violaceum* were distant from *P. officinale* but closely related to *Ligusticopsis*. Furthermore, the affinity between both species and *Ligusticopsis* was supported by the high similarity of their plastome sequences and also supported by the shared morphological features: stem base clothed in fibrous remnant sheaths, conspicuous calyx teeth, and strongly compressed dorsally mericarps with slightly prominent dorsal ribs, winged lateral ribs, and numerous vittae in the commissure and in each furrow [7,9,10]. However, hispid mericarps can easily distinguish *P. nanum* and *P. violaceum* from the glabrous mericarps of other *Ligusticopsis* species [7]. Moreover, *P. nanum* has densely hispid mericarps with slightly prominent dorsal ribs and six vittae in the commissure, whereas sparsely hispid mericarps with filiform dorsal ribs and eight vittae in the commissure are observed in *P. violaceum* [9,10]. Therefore, we could reasonably transfer *P. nanum* and *P. violaceum* into *Ligusticopsis* as two new members of this genus.

The sister relationship between *P. nanum* and *P. violaceum* was strongly supported by both the phylogenetic analyses based on plastome data and ITS sequences. The hispid mericarps shared by both species could further support this relationship [9,10]. Unfortunately, the relationship between this sister group and other *Ligusticopsis* species was not clearly resolved in our phylogenetic analyses. To confirm the phylogenetic position of the sister group of *P. nanum* and *P. violaceum* within *Ligusticopsis*, additional molecular sequences such as additional nuclear DNA fragments are required in future studies.

### 3.3. Taxonomic Treatment

*Ligusticopsis nana* (R.H.Shan and M.L.Sheh) C.K.Liu and X.J.He, comb. nov.

≡ *Peucedanum nanum* R.H.Shan and M.L.Sheh in Act. Phytotax. Sin. 18 (3): 377. 1980

Type: China. Xizang: Lhasa, in clivis montibus, 3500–3700 m, 16 September 1970, Kuo 8109 (holotype HNWP; isotype NAS!).

Distribution and habitat: This species is endemic to China (Xizang) and grows on dry mountain slopes with elevations of 3500–3800 m.

Additional specimens examined: China. Xizang: Rikaze, 3800 m, 1960, G.X. Fu 1377 (PE); Lhasa, 3821 m, 17 October 2021, J.J. Deng and R.X. Zhou LCK20211017-01 (SZ).

*Ligusticopsis violacea* (R.H.Shan and M.L.Sheh) C.K.Liu and X.J.He, comb. nov.

≡ *Peucedanum violaceum* R.H.Shan and M.L.Sheh in Act. Phytotax. Sin. 18 (3): 378. 1980

Type: China. Xizang: Mainling Xian, in locis arenosis montis Do Hsium, 2980 m, 11 August 1975, Qinghai-Xizang Exped. 751,309 (holotype PE; isotype KUN!).

Distribution and habitat: This species is endemic to China (Xizang) and occurs in sparse forests or grassy places on river banks with elevations of 2100–3500 m.

Additional specimens examined: China. Xizang; Lhoka City, Zhanang County, 3788 m, 25 August 2017, PE-Xiang Expedition PE5120 (PE); Nyingchi City, Mainling Country, 3100 m, 21 July 1972, Tibet Chinese Herbal Medicine Survey Team 3845 (PE); 3013 m, 16 September 2017, PE-Xiang Expedition PE6747 (PE); 2975 m, 19 October 2021, J.J. Deng and R.X. Zhou LCK2021101901 (SZ).

## 4. Materials and Methods

### 4.1. Plant Sample, DNA Extraction, Sequencing, and Assembly

Fresh leaves of *P. nanum* and *P. violaceum* were collected from their type localities and dried with silica gel. Voucher specimens were deposited in the herbarium of Sichuan University (Chengdu, China) (Table S1). Genomic DNA was extracted from the silica-gel-dried leaves using the modified CTAB method [57] and then fragmented into 400 bp to create a pair-end library according to the manufacturer’s protocol (Illumina, San Diego, CA, USA). Subsequently, the libraries were sequenced on the Illumina NovaSeq platform at Personalbio (Shanghai, China). The raw data yielded by Illumina sequencing were filtered with fastP v0.15.0 [58] to obtain high-quality reads with -n 10 and -q 15. The plastomes were then assembled based on the high-quality reads using NOVOPlasty v2.6.2 [59] with the default parameters and *rbc*L sequence extracted from the plastome of *L. rechingeriana* (MZ491175) as the seed. In addition, the ITS sequences were assembled using the GetOrganelle pipeline [60] with the ITS sequence of *L. rechingeriana* (MZ497220) as the reference.

### 4.2. Plastome Annotation and Feature Analyses

The assembled plastomes were initially annotated with the web server CPGAVAS2 (http://www.herbalgenomics.org/cpgavas2, accessed on 11 September 2022) [61]. Then, the start and stop codons and intron positions were manually corrected using Geneious v9.0.2 [62]. Finally, the online program OrganellarGenomeDRAW (OGDRAW) [63] was used to display the well-annotated plastomes.

Eleven plastomes of *Ligusticopsis*, which we have previously reported, were downloaded from the NCBI database (Table S8). In conjunction with two newly sequenced plastomes, codon usage of the thirteen plastomes was detected using CodonW v1.4.2 (Nottingham, UK). Subsequently, the potential RNA editing sites of the protein-coding genes for the thirteen plastomes were predicted using the online program Predictive RNA Editor for Plants suite with a cutoff value of 0.8 [64]. We also detected simple sequence repeats (SSRs) in the thirteen plastomes using MISA (http://pgrc.ipk-gatersleben.de/misa/, accessed on 11 September 2022). The minimum number of repeat units for mono-, di-, tri-, tetra-, penta-, and hexa-nucleotides was set to 10, 5, 4, 3, 3, and 3, respectively.

### 4.3. Comparative Plastome Analyses

The borders of the inverted repeat regions for the thirteen plastomes were compared in Geneious v9.0.2 [62]. Then, the gene order and sequence identity among the thirteen plastomes were investigated by using Mauve Alignment [65] implemented in Geneious v9.0.2 [62] and the mVISTA tool [66], respectively. To identify the mutation hotspot regions, the protein-coding genes, non-coding regions, and intron regions of the thirteen plastomes were extracted in Geneious v9.0.2 [62] and aligned with MAFFT v7.221 [67]. Alignments with more than 200 bp in length were used to calculate nucleotide diversity (Pi) using DnaSP v5.0 [68].

### 4.4. Phylogenetic Analyses

To resolve the phylogenetic positions of *P. nanum* and *P. violaceum*, 48 plastomes and 48 ITS sequences were used to reconstruct the phylogenetic tree (Tables S1 and S8). Among them, *Chamaesium mallaeanum* Farille and S.B.Malla and *Chamaesium viridiflorum* (Franch.) H.Wolff ex R.H.Shan were chosen as the outgroup based on a previous study [39]. The two datasets were aligned using MAFFT v7.221 [67]. Alignments were used for the maximum-likelihood analyses (ML) and Bayesian inference (BI). For the ML analyses, RAxML v8.2.8 [69] was used to reconstruct the phylogenetic tree with 1000 replicates and the GTRGAMMA model as suggested by the RAxML manual. The BI analyses were performed using MrBayes v3.2.7 [70]. The best-fit substitution models for plastome data (TVM+I+G) and ITS data (SYM+I+G) were tested using Modeltest v3.7 [71]. Two independent Markov chains were run for 1,000,000 generations with sampling every 100 generations and discarding the first 25% of the trees as burn-in.

## 5. Conclusions

The whole plastomes of *P. nanum* and *P. violaceum* were reported for the first time in the present study. The plastome comparisons among *P. nanum*, *P. violaceum*, and eleven *Ligusticopsis* species revealed that these plastomes were highly conserved and similar in terms of structure, size, GC content, gene content and order, IR borders, and the patterns of codon bias, RNA editing, and SSR. Nevertheless, 12 mutation hotspot regions (*mat*K, *ndh*C, *rps*15, *rps*8, *ycf*2, *ccs*A-*ndh*D, *pet*N-*psb*M, *psb*A-*trn*K, *rps*2-*rpo*C2, *rps*4-*trn*T, *trn*H-*psb*A, *ycf*2-*trn*L) were identified, which could serve as potential DNA markers for species identification and phylogenetic analysis of *Ligusticopsis*. Moreover, the phylogenetic analyses based on plastome data and ITS sequences robustly supported that *P. nanum* and *P. violaceum* nested in the genus *Ligusticopsis*. Considering also the morphological affinities, we transferred *P. nanum* and *P. violaceum* into *Ligusticopsis* and proposed two new combinations.

## Figures and Tables

**Table 1:** Comparison of plastome features among *Peucedanum nanum*, *P. violaceum*, and *Ligusticopsis* species.

Taxon	Total Length (bp)	LSC (bp)	SSC (bp)	IR (bp)	Total GC Content (%)	Total Genes (Unique)	Protein Coding Genes (Unique)	rRNA Genes (Unique)	tRNA Genes (Unique)
*L. brachyloba*	148,633	92,265	17,588	19,390	37.40%	113	79	4	30
*L. capillacea*	147,808	91,907	17,503	19,199	37.50%	113	79	4	30
*L. daucoides*	148,078	91,666	17,582	19,415	37.40%	113	79	4	30
*L. hispida*	147,797	91,846	17,627	19,162	37.40%	113	79	4	30
*L. integrifolia*	148,196	92,305	17,575	19,158	37.50%	113	79	4	30
*L. involucrata*	147,752	91,782	17,560	19,205	37.40%	113	79	4	30
*L. modesta*	148,133	92,247	17,568	19,159	37.50%	113	79	4	30
*L. oliveriana*	148,175	92,273	17,534	19,184	37.50%	113	79	4	30
*L. rechingeriana*	148,525	91,813	17,654	19,529	37.30%	113	79	4	30
*L. scapiformis*	148,107	92,214	17,581	19,156	37.50%	113	79	4	30
*L. wallichiana*	148,594	92,281	17,567	19,373	37.40%	113	79	4	30
*P. nanum*	146,900	91,480	17,308	19,056	37.50%	113	79	4	30
*P. violaceum*	148,190	91,811	16,335	20,022	37.50%	113	79	4	30

## Data Availability

Plastomes and ITS sequences of *P. nanum* and *P. violaceum* generated in the current study are available at the NCBI database (https://www.ncbi.nlm.nih.gov, accessed on 19 December 2022).

## Supplementary Materials

The supporting information can be downloaded at: https://www.mdpi.com/article/10.3390/ijms24010382/s1.
