# Supplementary material for: Plastid Phylogenomics Provide Evidence to Accept Two New Members of Ligusticopsis (Apiaceae, Angiosperms)
Source: Int J Mol Sci. 2022 Dec 26;24(1):382. doi: 10.3390/ijms24010382 (PMC9820081; doi:10.3390/ijms24010382)
Supplement: Supplementary file 1 [file ijms-24-00382-s001.zip › Figure S1.pdf]

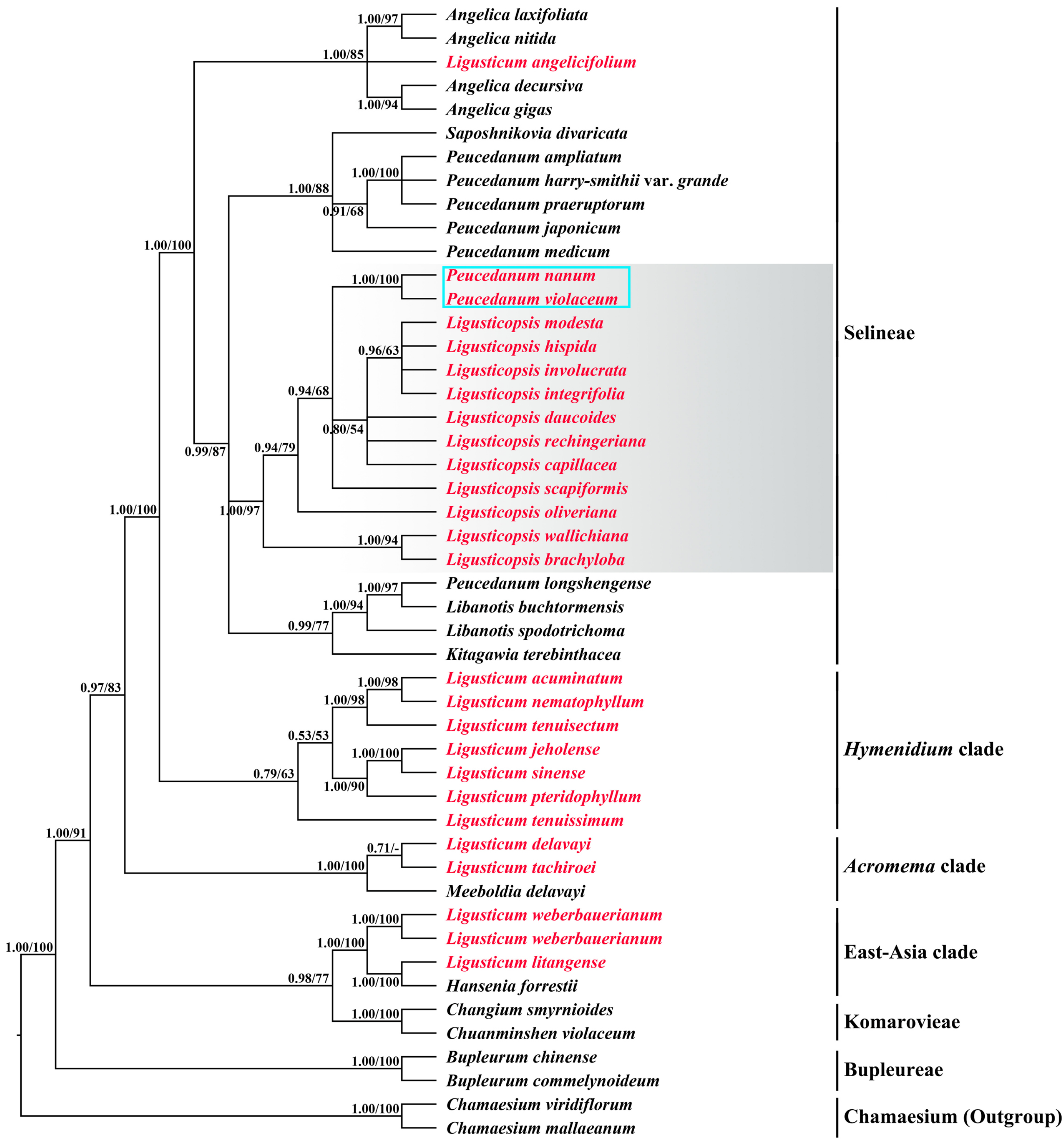

Figure S1. Phylogenetic tree inferred from Maximum likelihood (ML) and Bayesian inference (BI) analyses based on ITS sequences. Numbers indicate Bayesian posterior probabilities (PP) and maximum likelihood bootstrap values (BS), respectively.
