# Supplementary material for: Plastid Phylogenomics Provide Evidence to Accept Two New Members of Ligusticopsis (Apiaceae, Angiosperms)
Source: Int J Mol Sci. 2022 Dec 26;24(1):382. doi: 10.3390/ijms24010382 (PMC9820081; doi:10.3390/ijms24010382)
Supplement: Supplementary file 1 [file ijms-24-00382-s001.zip › Table S2.pdf]

Table S2. List of unique genes identified in plastomes of *P. nanum* and *P. violaceum.*

Category of Genes	Group of gene	Name of gene
Self-replication	Ribosomal RNA genes	*rrn*4.5, *rrn*5, *rrn*16, *rrn*23
	Transfer RNA genes	*trn*C-GCA, *trn*D-GUC, *trn*E-UUC, *trn*F-GAA, *trn*G-GCC, *trn*G-UCC*, *trn*H-GUG, *trn*I-CAU, *trn*K-UUU*, *trn*L-CAA, *trn*L-UAA*, *trn*L-UAG, *trn*M-CAU, *trn*P-UGG, *trn*Q-UUG, *trn*R-UCU, *trn*S-GCU, *trn*S-GGA, *trn*S-UGA, *trn*T-UGU, *trn*T-GGU, *trn*V-GAC, *trn*V-UAC*, *trn*Y-GUA, *trn*W-CCA, *trnf*M-CAU, *trn*A-UGC*, *trn*I-GAU*, *trn*N-GUU, *trn*R-ACG
	Ribosomal protein (small subunit)	*rps*2, *rps*3, *rps*4, *rps*7, *rps*8, *rps*11, *rps*12**, *rps*14, *rps*15, *rps*16*, *rps*18, *rps*19
	Ribosomal protein (large subunit)	*rpl*2*, *rpl*14, *rpl*16*, *rpl*20, *rpl*22, *rpl*23, *rpl*32, *rpl*33, *rpl*36
	RNA polymerase	*rpo*A, *rpo*B, *rpo*C1*, *rpo*C2
	Translational initiation factor	*inf*A
Genes for photosynthesis	Subunits of photosystem I	*psa*A, *psa*B, *psa*C, *psa*I， *psa*J, *ycf*3**, *ycf*4
	Subunits of photosystem II	*psb*A, *psb*B, *psb*C, *psb*D, *psb*E, *psb*F, *psb*H, *psb*I, *psb*J, *psb*K, *psb*L, *psb*M, *psb*N, *psb*T, *psb*Z
	Subunits of cytochrome	*pet*A, *pet*B*, *pet*D*, *pet*G, *pet*L, *pet*N
	Subunits of ATP synthase	*atp*A, *atp*B, *atp*E, *atp*F*, *atp*H, *atp*I
	Large subunit of Rubisco	*rbc*L
	Subunits of NADH dehydrogenase	*ndh*A*, *ndh*B*, *ndh*C, *ndh*D, *ndh*E, *ndh*F, *ndh*G, *ndh*H, *ndh*I, *ndh*J, *ndh*K
Other genes	Maturase	*mat*K
	Envelope membrane protein	*cem*A
	Subunit of acetyl-CoA	*acc*D
	Synthesis gene	*ccs*A
	ATP-dependent protease	*clp*P**
	Component of TIC complex	*ycf*1
Genes of unknown function	Conserved open reading frames	*ycf*2

*: Gene with one intron

**: Gene with two introns.
